# Challenges and Outcome of Management of Gastroschisis at a Tertiary Institution in North-Eastern Nigeria

**DOI:** 10.3389/fsurg.2020.00008

**Published:** 2020-03-04

**Authors:** Adewale O. Oyinloye, Auwal M. Abubakar, Samuel Wabada, Lateef O. Oyebanji

**Affiliations:** ^1^Division of Pediatric Surgery, Department of Surgery, Federal Medical Center, Yola, Nigeria; ^2^Division of Pediatric Surgery, Department of Surgery, University of Maiduguri Teaching Hospital, Maiduguri, Nigeria

**Keywords:** gastroschisis, resuscitation, improvised silo, primary closure, enteral feeding

## Abstract

**Introduction:** Gastroschisis is a congenital anterior abdominal wall defect characterized by herniation of abdominal contents through a defect usually located to the right side of the umbilical cord. It occurs in about 1 in 2,000–4,000 live births and is slightly commoner in males. Management has remained challenging in the low and middle-income countries, with high mortality rates. This study highlights the clinical presentation, treatment, outcomes, and challenges in the management of gastroschisis at a tertiary healthcare center in a resource-limited setting.

**Methods:** This was a retrospective review of the records of all patients with gastroschisis managed over a period of 30 months (January 2016–June 2018). Data on patients' demographics, age, birth weight, clinical presentation, method of gastroschisis reduction and closure, complications, and outcomes were collated. Statistical analysis was performed using SPSS version 20. A *p* < 0.05 was considered significant.

**Results:** Twenty-four patients with gastroschisis were managed. Of these, 18 patients had data available for analysis. There were 14 males, with a male-female ratio of 3.5:1. The median age at presentation was 11.0 h (range 1–36 h). Ten patients (55.6%) were delivered in a medical facility. One patient had type II jejunal atresia and transverse colonic atresia as associated anomalies. Improvised silos were applied by the bedside in 15 (83.3%) patients, while two patients (11.1%) had primary closure under general anesthesia. One patient died before definitive treatment could be done. Sterile urobags and female condoms were used for constructing improvised silos in 9 (60%) and 6 (40%) patients, respectively. Eight patients who had initial silo application had complete bowel reduction over a median time of 8.0 days (mean 10.0 ± 6.5days, range 2–23 days). Total parenteral nutrition was not available. The average time to commencement of feeding was 8.0 days ± 6.6 (median 6.0 days, range 2–22 days). Full feeding was achieved in five patients (two patients in the primary closure group and three from the silo group) over a mean time of 16.8 days ± 10.4 (median 14.0 days). Sepsis was the commonest complication. Four patients (22.2%) survived.

**Conclusion:** Management of gastroschisis remains challenging in resource-limited regions.

## Introduction

Gastroschisis is a congenital anterior abdominal wall defect characterized by herniation of abdominal contents through a defect usually located to the right side of the umbilical cord (1). In the last three decades, there has been a steady rise in incidence to a recent estimate of 1 in 2,000–4,000 live births (2–5). Management has remained challenging in the low and middle-income countries (LMICS), with reported mortality rates ranging between 33 and 100% (6–12). By contrast, survival rates in high-income countries are above 95% (4, 5).

This study highlights the clinical presentation, treatment, outcomes, and challenges in the management of gastroschisis in our setting.

## Methods

This was a retrospective review of the records of all patients with gastroschisis managed in our institution over a period of 30 months (January 2016–June 2018). Ethical approval was obtained from the hospital health research ethics committee (approval number FMCY/HREC/19/54). Data on patients' demographics, age, birth weight, clinical presentation, method of gastroschisis reduction and closure, complications, and outcomes were collated. Statistical analysis was performed using SPSS version 20 (Chicago, Illinois). Continuous variables were compared with Student *t*-test and discrete variables were analyzed with Chi-square and Fischer exact test where applicable. Results were presented in the form of means, standard deviations, median and percentages. A *p* < 0.05 was considered significant.

## Results

Twenty-four patients with gastroschisis were managed during the period. Of these, 18 patients had data available for analysis. There were 14 males, with a male-female ratio of 3.5:1. The median age at presentation was 11.0 h (mean 12.6 h ±9.6 h, range 1–36 h). The mean weight on admission was 2.1 kg± 0.4 (median 2.2 kg, range 1.4–3 kg). All patients were born per vaginal delivery.

Ten patients (55.6%) were delivered in a medical facility (primary health care centers, secondary health care centers, and private clinics), while 8 (44.4%) were delivered at home. All the patients who delivered in a healthcare center attended at least one antenatal clinic visit. However, none of the patients had a prenatal diagnosis before presentation.

The anterior abdominal wall defect was located on the right side of the umbilical cord in all patients, with a mean diameter of 3.1 ± 1.4 cm (range 1–5 cm) recorded in 13 patients. The defect size was not stated in five patients. Three of the patients arrived with a temperature below 36°C, while three patients had temperatures above 37.5°C.

## Associated Anomalies

There were two associated anomalies found in one patient (5.9%) who had type II jejunal atresia and transverse colonic atresia. Intestinal perforation unrelated to intestinal atresia was recorded in one patient.

## Treatment Method

**Figure 1:**
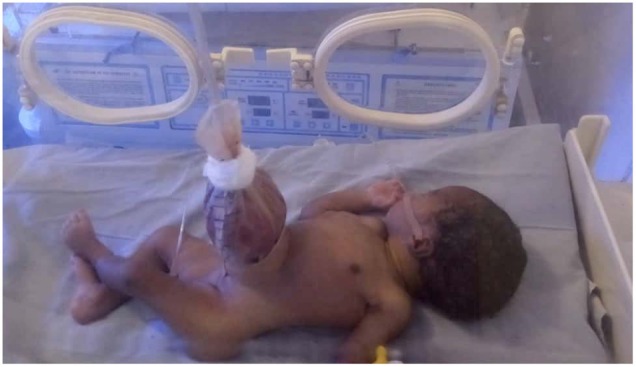
Urobag improvised as silo in a neonate with gastroschisis.

Improvised silos (Figure 1) were applied by the bedside in 15 (83.3%) patients, while two patients (11.1%) had primary closure under general anesthesia in theater. The decision to do primary closure was based on the assessment of the size of defect and the condition of herniated bowel at presentation. The two neonates with smaller defects presenting early before significant bowel edema developed underwent primary closure. One patient died before definitive treatment could be done. Sterile urobags and female condoms were the materials used for constructing improvised silos in 9 (60%) and 6 (40%) patients, respectively. After skin preparation with antiseptic solution, skin, and fascia around the defect were infiltrated with local anesthetic (3 mg/kg of 1% xylocaine) and intravenous analgesic (paracetamol 10 mg/kg body weight) was administered before the procedure. The silos were then placed over the bowel and anchored to abdominal wall with sutures. Three patients had silo disruptions (two female condoms, one urobag) on two or more occasions, necessitating re-application. Eight patients who had initial silo application had complete bowel reduction over a median time of 8.0 days (mean 10.0 ± 6.5 days, range 2–23 days). However, only six eventually had delayed closure as the other two patients died from sepsis and suspected bowel ischemia shortly before closure.

## Commencement of Feeding

Following successful gastroschisis reduction and closure, oral feeding was introduced in the two patients who had primary closure and in six patients who had initial silo and delayed closure. Feeding was generally commenced at about 10–20 mls/kg/days and increased depending on how well the babies tolerated the feeds. Some babies tolerated feeds faster than others, so the feeding protocol was individualized and gradually increased as tolerated by the neonates. The intravenous fluid requirements was tailed down accordingly. The average time to commencement of feeding was 8.0 days ±6.6 (median 6.0 days, range 2–22 days). Full feeding was achieved in five patients (two patients in the primary closure group and three from silo group) over a mean time of 16.8 days± 10.4 (median 14.0 days, range 4–32 days). Total Parenteral Nutrition (TPN) was not available.

## Complications and Outcome

There were some complications seen during the management of these patients. These complications include clinical features of sepsis in 18 (100%), malnutrition (weight loss/poor weight gain) in seven (44.4%), anemia in 6 (38.9%), and surgical site infection (SSI) in three patients. Blood culture yielded growth of Klebsiella in three patients. Electrolyte imbalances were recorded in 5 patients, while coagulopathy was seen in one patient. One patient had minor wound dehiscence post closure. The median duration of hospital stay in the children that survived was 29 days (mean 29.3 days), compared to 6.2 days (mean 11.8 days) in those that died (*p* = 0.02). The overall median duration of hospital stay in all the patients was 11.5 days (mean 15.7 days± 13.8, range 2–45 days).

Four patients (22.2%) survived, giving a mortality rate of 77.8%. Of these, three had initial silo application and delayed closure while one had primary closure. There was no significant difference in the outcome between primary closure and silo plus delayed closure groups (*p* = 0.55).

There was no significant difference between the average age at presentation among survivors (12.75 h ± 10.0) and those that died (12.50 ± 9.21). Although the mean weight of survivors (2.43 kg ± 0.59) was higher than that of the neonates who died (1.99 ± 0.34 kg), this difference was not statistically significant (*p* = 0.07).

Overwhelming sepsis (mostly a clinical diagnosis) was responsible for mortality in 11 patients (78.6%). One patient each died from bowel ischemia (suspected NEC), anemic heart failure, and aspiration pneumonitis. There was no statistically significant association between the place of delivery and outcome (*p* = 0.09) although the four survivors were born in healthcare centers while all babies delivered outside a healthcare facility died.

## Discussion

There has been a rising incidence of gastroschisis worldwide in the last 3 decades (3–5). In a 2012 survey carried out among pediatric surgeons by Wright and colleagues, an estimated average of 22 cases per institution per year was reported in low-income countries, while middle- income and high-income countries reported 12 and 15 cases per institution per year, respectively (13). This study identified 24 cases over a 30 months period with an average of about 9 cases per year. By contrast, Ameh et al. in the year 2000 reported 14 cases of gastroschisis over 10 years at a tertiary hospital in Zaria, northern Nigeria (8). Similarly, in 2003, Uba and Chirdan identified 12 patients with gastroschisis in Jos teaching Hospital, north-central Nigeria between 1991 and 2001 (14). Although there are no formal congenital anomaly registers in Nigeria, it would appear that our study is reflective of the rising incidence of gastroschisis worldwide. It could also be due to increasing awareness in the community, resulting in more patients presenting for care in tertiary facilities. However, further epidemiological studies need to be done to accurately determine the incidence of gastroschisis in our region and the nation as a whole.

Delayed presentation to tertiary pediatric surgery center is a major problem in the management of gastroschisis in low resource settings. In this study, the majority of the patients (55.6%) were delivered in primary or secondary level health centers. None of the patients had a prenatal diagnosis and the mean age at presentation to our facility was 12.6 h. As a result, all the neonates were delivered outside a tertiary health center (at home, primary healthcare clinics, secondary health care centers, and private hospitals) that can manage neonates with gastroschisis. Often, these babies are transported without adequate initial resuscitative care, usually over long distances. We observed that about one-third of our patients had fever or sub-normal temperatures on arrival. In Kampala Uganda, Wesonga et al. similarly observed that 81% of neonates with gastroschisis were born in first or second level healthcare centers, without appropriate care being initiated, and only 58% arrived within 12 h of delivery (15). The role of initial resuscitative care and transport of the surgical neonate cannot be over-emphasized. A study by Stevens and colleagues showed that poor resuscitation is a more significant predictor of mortality than postnatal transfer time, further highlighting the impact of adequate initial care for babies with gastroschisis (16). Although not statistically significant in this study, delay in transfer time, coupled with the attendant poor initial medical care may have had a role to play in the eventual outcome.

In our study, 10 patients had antenatal ultrasound scanning at least once during pregnancy. However, prenatal diagnosis was not made. This is similar to the finding of Abdur-Rahman et al. who reported that despite the availability of antenatal ultrasound scanning in Ilorin, Nigeria, only one of seven cases of gastroschisis was diagnosed prenatally (12). The World Health Organization (WHO) guidelines on antenatal care in pregnancy has been largely adapted and implemented for a significant proportion of pregnant women in low and middle-income countries (17). However, ultrasound scanning is currently not recommended as part of the antenatal care package (17). In our environment, we have observed that ultrasound often focuses on basic obstetrics parameters and evidence of fetal viability with little attention paid to the detection of congenital anomalies. The varying levels of reliability of some of the antenatal scans have also been identified as a potential problem (15). We believe that improvement in prenatal diagnosis will improve the outcome, as proper planning for delivery and prompt post partum care can be made.

The majority of our patients had improvised silo application by the bedside as the initial modality of treatment. This was because most babies were not fit for closure under general anesthesia as the immediate post-admission care was centered around proper resuscitation. Also, the presence of significant bowel edema and concomitant risk of bowel ischemia/compartment syndrome precluded attempts at primary closure. In addition to these, we do not have the capacity for neonatal mechanical ventilation and studies have shown that the use of preformed silos is associated with lower requirements for ventilation (18–20), reduced time to feeding, lower infection rates and lower risk of abdominal compartment syndrome (20). However, preformed silos are not available in our setting, so we improvised using the female condom initially and switched to urobag subsequently, because urobag is stronger and does not tear easily like the condom. The improvised silos have its associated risks and impact on the outcome. Hasan et al. used sterile saline bags or urobags for silo reconstruction and observed that almost all the patients (39/40) developed sepsis and had poor outcomes (21). An international survey identified likely reasons for the limited use of preformed silos in low and middle-income countries to include the lack of availability, expertise, and expense (13).

Non-availability of parenteral nutrition was a significant challenge encountered in this study. When available, it was in the form of an expensive amino acid infusion (astymin® and similar brands) in adult-sized bottles ideally meant to be utilized within 24 h once opened. The cost of each bottle is about 3,500–5,500 Nigerian Naira (10–15 US Dollars). Occasionally, we have had cause to improvise by sharing the bottle contents into different burettes for administration to babies who may benefit from it. An international survey reported that only 19% of tertiary pediatric surgery centers in low-income countries had access to parenteral nutrition (13). Wright and colleagues rightfully noted that the challenges to the provision of parenteral nutrition in resource-limited settings include lack of infrastructure and availability of neonate-specific parenteral nutrition bags, difficulty in achieving central venous access and shorter bench life of neonatal parenteral nutrition where available (22).

Commencement of enteral feeding was achieved in eight of our patients over an average time of 8 days (median 6 days). Early enteral feeding has been proven to be beneficial in patients with gastroschisis (23, 24). This will be especially important in our setting where parenteral nutrition is not readily available. A systematic review showed that delay in commencement of enteral feeds was associated with a longer period to full enteral feeding, duration of parenteral nutrition and duration of hospital stay (24). Because we are aware of our limitations with parenteral nutrition, we usually aim to achieve closure early and start gradual enteral feeding. The longest time to commencement of feeding was 22 days post-primary closure in the neonate with type II jejunal atresia and transverse colonic atresia. This was presumably due to the attendant dysmotility and slow return of bowel function usually seen with intestinal atresia. The patient, unfortunately, succumbed to sepsis on the 35th day of life.

Similar to many reports from Africa (6–12), the mortality recorded in this study was high. This may be connected to the delayed transfer to a tertiary healthcare facility, non-availability of parenteral nutrition and neonatal intensive care; factors that have been attributed to the improved survival rates in patients with gastroschisis in the developed world (7, 13, 25). Most of the deaths were due to clinically diagnosed sepsis, though this was not proven by positive culture results in most cases. Other deaths were as a result of preventable causes like severe anemia and aspiration pneumonitis. We have since taken pro-active measures to reduce the high mortality rates in patients with gastroschisis in our hospital. An antibiotic protocol has recently been put in place, and simple but effective measures like hand washing among staff members in between patients have been reinforced. The institution has stepped up plans to develop a pediatric and neonatal intensive care unit as well as engage in personnel training programs for health workers involved in the management of children within and outside our tertiary healthcare center. We are also currently enrolled in a multi-center, prospective evaluation of the management of gastroschisis and other congenital anomalies, the results of which would be useful in articulating policies for further improvement in the care of neonates with gastroschisis in our setting (26). We also acknowledge that this present study is limited by the relatively small sample size from a single centre and the retrospective nature of the research.

In conclusion, gastroschisis remains a challenging condition to manage in our region. Improvement in initial care of the neonate, prompt and proper conveyance to the point of surgical care will help reduce complications. The importance of neonatal intensive care facilities, parenteral nutrition, and appropriate personnel and material resources cannot be overemphasized.

## Data Availability Statement

The datasets generated for this study are available on request to the corresponding author.

## Ethics Statement

The studies involving human participants were reviewed and approved by Health Research Ethics Committee, Federal Medical Center, Yola, Adamawa State, Nigeria. Written informed consent to participate in this study was provided by the participants' legal guardian/next of kin.

## Author Contributions

AO and AA conceptualized the study. AO, SW, and LO were involved in study design, data collation, analysis, interpretation of results, and initial draft of manuscript. AO, AA, and SW reviewed the final manuscript.

### Conflict of Interest

The authors declare that the research was conducted in the absence of any commercial or financial relationships that could be construed as a potential conflict of interest.
