# Erratum: Challenges and Outcome of Management of Gastroschisis at a Tertiary Institution in North-Eastern Nigeria

**DOI:** 10.3389/fsurg.2020.00015

**Published:** 2020-03-24

**Authors:** Frontiers Production Office

**Affiliations:** Frontiers Media SA, Lausanne, Switzerland

**Keywords:** gastroschisis, resuscitation, improvised silo, primary closure, enteral feeding

Due to an editorial error, an outdated version of the manuscript was published. The publisher apologizes for this mistake.

The correct article version has now been published.

